# Effects of Dietary Inclusion of β-Hydroxy-β-Methylbutyrate on Growth Performance, Fat Deposition, Bile Acid Metabolism, and Gut Microbiota Function in High-Fat and High-Cholesterol Diet-Challenged Layer Chickens

**DOI:** 10.3390/cimb44080235

**Published:** 2022-07-30

**Authors:** Qichao Liao, Tian Wu, Qinghua Fu, Peng Wang, Yameng Zhao, Yan Li, Haihan Xiao, Lei Zhou, Ziyi Song

**Affiliations:** State Key Laboratory for Conservation and Utilization of Subtropical Agro-Bioresources, College of Animal Science and Technology, Guangxi University, Nanning 530004, China; qc.liao@foxmail.com (Q.L.); wutian980518@126.com (T.W.); fuqinghua.good@163.com (Q.F.); wangpeng131477@126.com (P.W.); z152zgn@163.com (Y.Z.); lyyouth0305@163.com (Y.L.); xiaohaihan0825@163.com (H.X.); zhoulei@gxu.edu.cn (L.Z.)

**Keywords:** layer chickens, fat deposition, β-hydroxy-β-methylbutyrate, bile acids, gut microbiota

## Abstract

Excessive lipid deposition in layer chickens due to inappropriate feeding adversely affects egg production; however, nutritional manipulation methods to deal with this issue are still limited. β-hydroxy-β-methylbutyrate (HMB), a metabolite of L-leucine, was recently reported as a lipid-lowering nutrient in mice and pigs, although its role in layers had not been investigated. Here, we employed high-fat and high-cholesterol diet (HFHCD)−challenged growing layers as an obese model to explore HMB function in the regulation of lipid metabolism and the potential mechanisms involved. We found that dietary supplementation with (0.05% or 0.10%) HMB significantly reduced HFHCD−induced bodyweight growth in layers, mainly due to reduction in abdominal fat deposition. Mechanistically, HMB supplementation enhanced hepatic bile acid synthesis from cholesterol through elevating expression of *Cyp7a1*, a gene coding a key enzyme in bile acid synthesis. Furthermore, 16S rRNA gene sequencing revealed that HMB supplementation remodeled the diversity and composition of the layers’ cecal microbiota, and the abundance of *Bacteroidetes* at the phylum level were especially affected. Correlation analysis further indicated a strong negative association between *Bacteroidetes* abundance and lipid metabolism−related parameters. Taken together, these data suggest that dietary HMB supplementation could improve abdominal fat deposition in layers, probably through modulating hepatic bile acid synthesis and gut microbiota function.

## 1. Introduction

The lipid deposition and health status of egg–laying hens is crucial to the development of the poultry industry [1]. Due to continuous egg production and a high dietary consumption of carbohydrates, excessive fat deposition is a common issue in layers, which results in increased risk of developing several metabolic disorders, including insulin resistance, hepatic inflammation, and even fatty liver haemorrhagic syndrome (FLHS) [2,3,4,5]. These metabolic disorders further lead to abnormal ovarian morphology, poor egg production, and high mortality in layer chickens, and ultimately cause major economic losses for the poultry industry [1]. In addition, studies have shown that the metabolic disorders in layers are similar to humans’ [4]; therefore, studies trying to ameliorate layer fat accumulation in hens not only benefit the poultry industry, but also could give insights into the treatment of human obesity. 

Generally, excessive deposits of fat in layers, especially in the abdomen, results from an imbalance in lipid homeostasis in adipose tissue: the rate of fat acquisition through fatty acid uptake and *de novo* lipogenesis (DNL) surpasses that of disposal via fatty acid oxidation and export of lipids [6,7,8]. Notably, in chickens, the liver is the primary site for lipid metabolism, where more than 90% of *de novo* fatty acids are synthesized [9,10]. The synthesized lipids are packaged as lipoproteins, mainly very low-density lipoproteins (VLDLs) and vitellogenin (Vg), which are secreted into the blood stream and taken up by adipose tissue for long-term storage or by growing oocytes for egg yolk formation [7,11]. Therefore, direct or indirect inhibition of hepatic DNL or promotion of hepatic lipid oxidation is considered as a strategy to reduce fat accumulation in laying hens. In addition, a large body of studies on birds and mammals demonstrate that gut microbiota play a dramatic role in the regulation of host lipid metabolism through modulating the intestinal barrier and inflammation by the production of metabolites, such as short-chain fatty acids (SCFAs) [12,13]. Dysbiosis of the gut microbiota, defined as a decrease in commensal bacteria levels and diversity, has been linked to obesity and fatty liver both in chicken and humans. Thus, manipulation of gut microbiota diversity and composition is another way of dealing with obesity [12,14]. However, despite increasing understanding of the factors driving fat over-accumulation in laying hens, low-cost drugs that are preferable for doctors and animal producers are still limited.

β-Hydroxy-β-methylbutyrate (HMB) is a derivative of leucine and is metabolized in the liver from the keto acid of leucine by α-ketoisocaproate dioxygenase [15]. Studies have shown that HMB can be used as a nutritional supplement to exert positive effects in animals and humans under stressful or inflammatory conditions [16,17,18]. Specifically, recent studies have indicated that low-dose dietary HMB supplementation alleviates dorsal subcutaneous fat deposition in pigs and global obesity in high-fat diet (HFD)-induced obese mice though remodeling of gut microbiota composition and function, especially *Bacteroidetes*−mediated SCFA production [19,20]. Moreover, studies on broiler chickens have revealed that 0.10% HMB improves growth performance, meat quality, and hepatic lipid accumulation [21,22]. However, until now, there have been no reports regarding HMB in laying hens. To this end, we employed high-fat and high-cholesterol diet (HFHCD)–fed growing Hy-line Brown as an obese model to explore the effects and underlying mechanisms of dietary HMB supplementation on growth performance and lipid metabolism in layer chickens. The findings of this study may contribute to overcoming the challenge of lipid metabolic diseases in layers during the late laying period.

## 2. Materials and Methods

### 2.1. Animals and Diet 

All animal studies were conducted according to protocols approved by the Animal Ethics Committee of Guangxi University (GXU-2022-018). One-hundred 1-day-old Hy-line Brown chickens were purchased from a live-poultry market (Nanning, China) and housed in an environmentally controlled room in the Guangxi University Animal Experimental Center. All birds were allowed free access to food and water. At 0~4 weeks, all layers were fed with standard starter feeds. After that, the birds were randomly assigned to four groups (3 replicates per group, 8−9 birds per replicate), and fed with one of four diets for 6 weeks: ND−CN (basic diet), HFHCD−CN (basic diet + 13% lard + 2% cholesterol), HFHCD−0.05% HMB (HFHCD + 0.05% HMB), and HFHCD−0.10% HMB (HFHCD + 0.10% HMB). The HMB (purity ≥ 97.0%, Y25001) was obtained from Yuanye Bio-Technology Co., Ltd. (Shanghai, China). The starter feeds and ND−CN diet were ordered from Guangxi Fufeng Agriculture and Animal Husbandry Corporation, Ltd. (Nanning, China), and the compositions of the diets are shown in Table 1.

### 2.2. Measurement of Growth Performance

For growth performance analysis, six birds were randomly selected from each replicate. Fasting weight was measured at 0, 3, and 6 weeks post−HMB supplementation. Feed intake was recorded weekly. Based on fasting weight and feed intake data, average daily feed intake (ADFI), feed-to-gain ratio (F/G), and average daily gain (ADG) were calculated following the standard formulas [23].

### 2.3. Slaughter Indicators and Organ Index

At the end of HMB supplementation for 6 weeks, the birds were slaughtered after 12 h fasting. Abdominal fat weight, extra-muscular fat weight, eviscerated weight, and intermuscular fat width were measured. Notably, intermuscular fat width refers to the width of the middle and lower fat bands between the axilla and sternum along the edge of pectoralis major, while percentage of abdominal fat (PAF) was calculated as follows: percentage of abdominal fat (%) = [(abdominal fat weight + extra-muscular fat weight)/eviscerated weight] × 100.

### 2.4. Measurement of Plasma and Hepatic Parameters

Total cholesterol (TC), triglyceride (TG), and total bile acid (TBA) contents in the plasma or liver tissues were all measured using assay kits (A111-1-1, A110-1-1, and E003-2-1; Nanjing Jiancheng Bioengineering Institute, China), according to the manufacturer’s instructions. Protein concentrations in tissue samples were determined using the BCA protein quantitative assay kit (Beyotime Biotechnology, Shanghai, China). TC and TG levels were expressed as mmol/g protein.

### 2.5. RNA Extraction and Quantitative Real-Time PCR

RNA was extracted from liver tissues by re-suspending 10−20 mg of frozen tissues in 1 mL Trizol (Life Technologies, Foster City, CA, US) followed by lysing for 5 min at 25 Hz with steel balls in a tissue lyser (Qiagen, Hilden, Germany). The total RNA was then extracted, according to the manufacturer’s directions for Trizol. Tecan Infinite^®^ M200 Pro Instrument (Tecan Austria GmbH, Grödig, Austria) was used to measure the purity and concentration of total RNA. The RevertAid First Strand cDNA Synthesis Kit (Thermo Fisher Scientific, MA, US) was used to make cDNA from 1 μg of total RNA. For real-time qPCR, 2× RealStar Green Fast Mixture was used (GenStar, Beijing, China). All data were adjusted to the amount of β-actin before being analyzed using the 2^−ΔΔ*Ct*^ approach. The sequences of primers used in this study are listed in Table 2.

### 2.6. Hematoxylin and Eosin (H&E) Staining

H&E staining was performed as described previously [24]. Briefly, the livers and adipose tissues of the layers were dissected and fixed in tissue-fixing solutions overnight at 4 °C. Then, samples were sent to Wuhan Service Technology Co., Ltd. (Wuhan, Hubei, China) for paraffin embedding, sectioning, and staining. Images were collected by light microscope (Biological microscope ML31; MSHOT, Guangzhou, China), and Image J software was used to examine the cell areas.

### 2.7. Gut Microbiome Analysis by 16S rRNA Sequencing

Gut microbiome analyses by 16S rRNA sequencing were performed as described previously [25]. Purified fecal microbiome DNA from the layer cecal samples were sent to the Beijing Tsingke Biotechnology Co., Ltd. (Beijing, China) for 16S rRNA V4 region amplification, and sequencing was performed using the Illumina Novaseq 6000 PE250 platform (Illumina, San Diego, CA, USA). The obtained sequence files were then analyzed using QIIME2 (v2021.2.0) in the following steps. First, the raw reads for each sample were normalized to yield 40,000 featured sequences. Then, an amplicon sequence variant table (ASV) was created after noise-reduction manipulation was carried out using the DADA2 module in QIIME2. After that, 16S rRNA genes were annotated based on the previously trained V4 region classifier in GreenGenes (https://greengenes.lbl.gov, accessed on 14 February 2022). Next, alpha-diversity indices (Shannon, Simpson, Chao1, and ACE) were applied to describe the diversity of microbiome groups among samples using Vegan (v2.5-7, R package). Beta diversity was assessed by Phyloseq (v1.3.20, R package). A Venn diagram was drawn by ggClusterNet (v0.2, R package). Taxa abundances for specific genera were summed at the phylum level and genus level by amplicon sequencing (v1.13.0, R package). To identify distinct groups in the multiple samples, the linear discriminant analysis (LDA) effect size (LEfSe) method and the non-parametric factors Kruskal–Wallis rank-sum test were used. PICRUSt (http://huttenhower.sph.harvard.edu/galaxy, accessed on 1 March 2022) was employed to predict the KEGG functions of microbial communities based on ASV tables. Pearson correlation analysis was performed to determine the correlations between fecal flora abundance and hepatic total bile acids, percentage of abdominal fat (PAF), and hepatic TG or TC levels. All the results were visualized using the R package ggplot2 (v3.3.5).

### 2.8. Statistical Analysis

The results are expressed as means ± SDs. Significance was estimated by unpaired Student’s *t*-test (for two groups) or one-way ANOVA (for multiple groups). A probability of *p* < 0.05 was considered to be statistically significant. The statistical analysis and figures were prepared using GraphPad Prism 8.0.

## 3. Results

### 3.1. Effects of Dietary HMB on the Growth Performance of HFHCD−Challenged Layer Chickens

**Figure 1:**
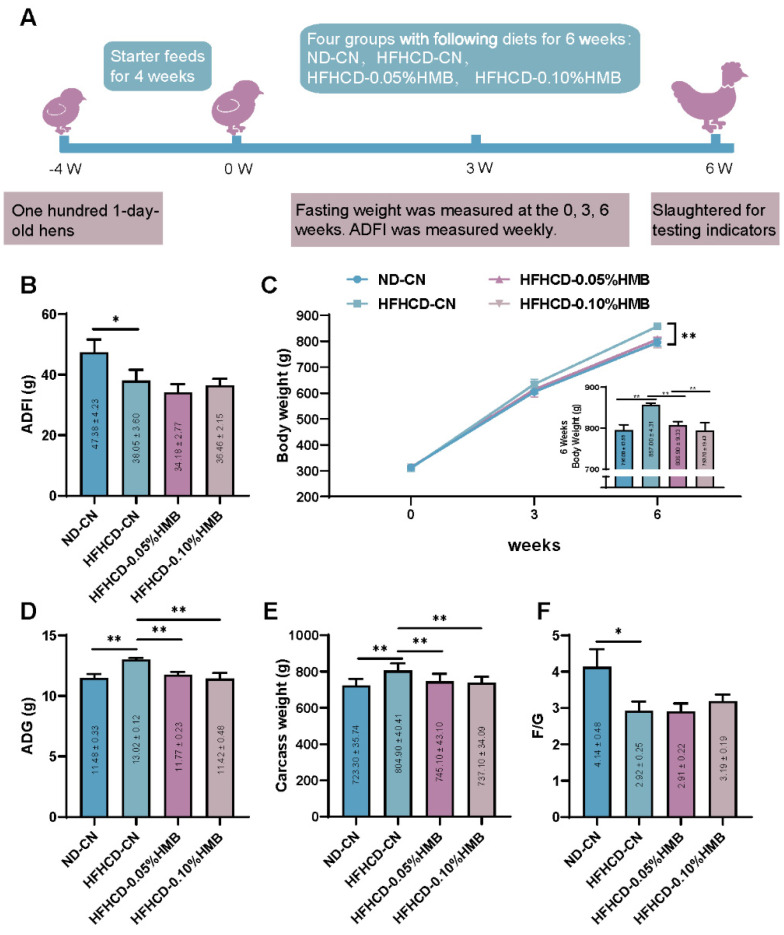
Effects of HMB supplementation on the growth performances of HFHCD−challenged layers. (**A**) Diagram of the experimental design. (**B**) Average daily feed intake. (**C**) Bodyweight curve of the growing hens. (**D**) Average daily gain weight. (**E**) Carcass weight of the growing hens (**F**) Feed-to-gain ratio of the growing hens. All the results are shown as the means ± SDs; *n* = 3; * *p* < 0.05, ** *p* < 0.01.

To facilitate the investigation of the role of HMB supplementation on lipid metabolism in layer chickens, we employed a high-fat and high-cholesterol diet (HFHCD)−induced fat over-accumulation hen model and monitored the effects of HMB supplementation on the growth performance of the birds during the full trial (Figure 1A). We found that, compared with the normal diet (ND), HFHCD feeding reduced the average daily feed intake (ADFI) of the birds regardless of supplementation with HMB or not (Figure 1B). Despite less feed intake, after 6 weeks of feeding, HFHCD significantly increased bodyweight of the layers compared with the control group, indicating that the HFHCD−induced obese model would be successful (Figure 1C). Importantly, 0.05% and 0.10% HMB supplementation obviously attenuated HFHCD−induced bodyweight growth, implying that HMB might alleviate HFHCD−induced fat deposition (Figure 1C). Consistent with the difference in gross bodyweight, HMB supplementation also decreased the HFHCD−induced average daily gain (ADG) and carcass weight of the birds (Figure 1D,E). Furthermore, we observed that, compared with the control group, feed-to-gain ratio (F/G) was decreased in the HFHCD group with or without HMB supplementation (Figure 1F), suggesting that HFHCD enhances the feed conversion rate in hens. Collectively, these data indicate that dietary HMB supplementation attenuates HFHCD−induced bodyweight growth in layer chickens.

### 3.2. Effects of Dietary HMB on HFHCD−Induced Lipid Accumulation in Layer Chickens

**Figure 2:**
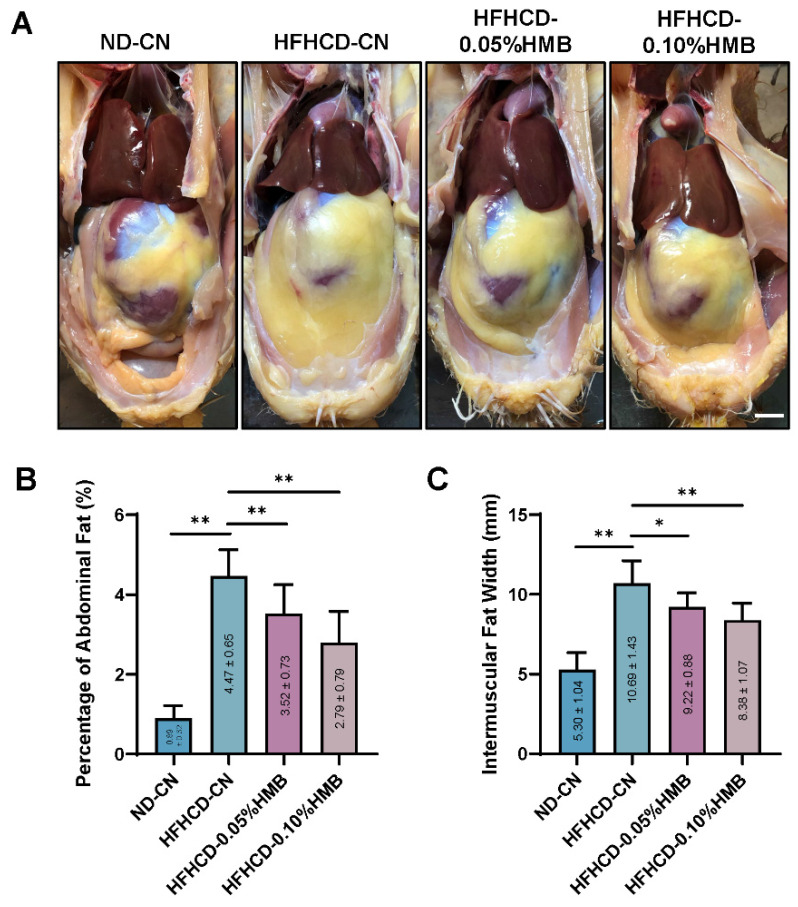
Effects of HMB on fat deposition in HFHCD−challenged layers. (**A**) Representative pictures of layers. Scale bar, 1 cm. (**B**) Percentage of abdominal fat (*n* = 10). (**C**) Intermuscular fat width (*n* = 10). All the results are shown as the means ± SDs; * *p* < 0.05, ** *p* < 0.01.

**Figure 3:**
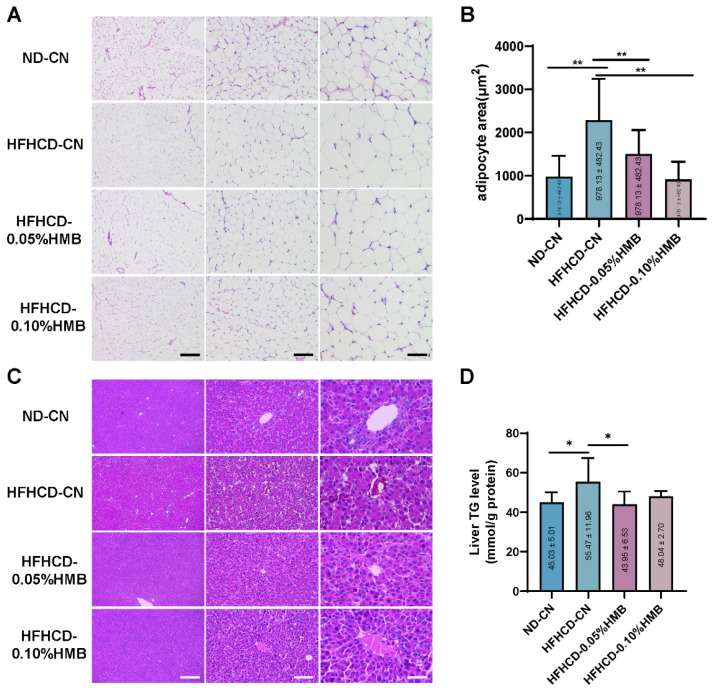
Effects of HMB on adipose and hepatic lipid content in HFHCD−challenged layers. (**A**) Representative images of abdominal adipose tissue with H&E staining, viewed under a microscope (40×, 100×, and 400×) (**B**) ImageJ analysis of fat cell area size according to panel A (*n* ≥ 90). (**C**) H&E staining of layer liver section, viewed under a microscope (40×, 100×, and 400×). (**D**) Liver triglycerides (TG) levels (*n* = 8). All the results are shown as the means ± SDs; * *p* < 0.05, ** *p* < 0.01.

Next, to determine whether HMB supplementation could reduce fat deposition in layers, the birds were dissected at the end of the trial. As shown in Figure 2A, the mass of abdominal fat was found to be markedly increased in the HFHCD−fed group compared with the ND−CN group, further demonstrating that the hen model of HFHCD−induced obesity was successful. However, it was also clear that 0.05% or 0.10% HMB supplementation significantly reduced the HFHCD−induced accumulation of abdominal fat (Figure 2A). Consistently, the quantitative analysis of abdominal fat index and intermuscular fat width further supported the observation that HMB supplementation ameliorated HFHCD−induced abdominal fat deposition (Figure 2B,C). Furthermore, adipose tissue histologic analysis showed that two levels of HMB supplementation dramatically reduced the HFHCD−induced increase in the cell size of abdominal fat (Figure 3A,B). By contrast, no obvious difference was observed in the liver section, although quantitative analysis showed slight alterations in hepatic triglyceride (TG) levels (Figure 3C,D). Collectively, these data demonstrate that dietary supplementation with HMB could mitigate HFHCD−induced abdominal fat deposition in hens.

### 3.3. Effects of Dietary HMB on Hepatic Bile Acid Metabolism in HFHCD−Challenged Layer Chickens

**Figure 4:**
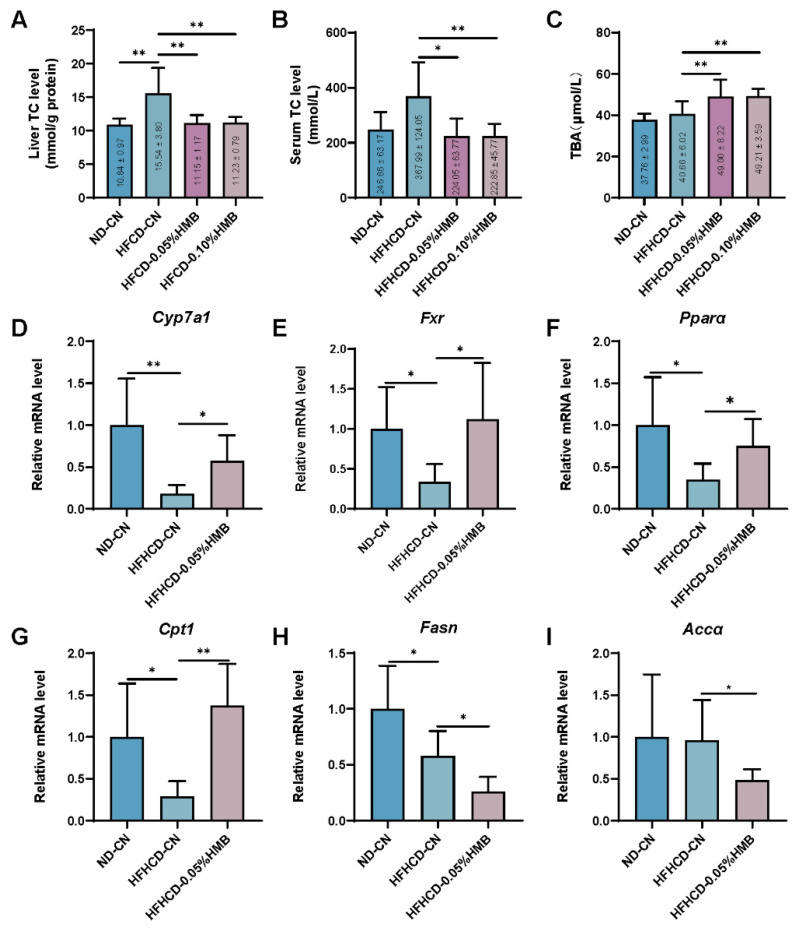
Effects of HMB on hepatic cholesterol and bile acid metabolism in HFHCD−challenged layers. (**A**) Liver total cholesterol (TC) content (*n* = 8). (**B**) Serum total cholesterol (TC) content (*n* = 6–8). (**C**) Liver total bile acid (TBA) content (*n* = 8). (**D**–**I**) qRT-PCR analysis of the levels of *Cyp7a1*, *Fxr*, *Pparα*, *Cpt1*, *Fasn*, and *Accα* (*n* = 5–6). All the results are shown as the means ± SDs; * *p* < 0.05, ** *p* < 0.01.

It is well-known that the liver, as a central metabolic organ, acts as a critical hub for numerous physiological processes, including the metabolism of glucose, lipids and cholesterol, and so forth, in mammals [26,27]. Given that the layers were challenged with HFHCD, next, we measured hepatic cholesterol levels. As expected, HFHCD feeding increased the cholesterol levels in the liver by 50% compared with the ND control (Figure 4A). However, unexpectedly, HMB supplementation significantly reduced HFHCD−induced hepatic cholesterol enrichment (Figure 4A). In line with hepatic cholesterol levels, a similar result was observed for serum cholesterol levels (Figure 4B). Then, to reveal how HMB affects cholesterol metabolism, we checked the levels of hepatic total bile acids, since cholesterol can be removed through bile acid synthesis [28]. As shown in Figure 4C, we found that there was a significant increase in total bile acid levels after HMB treatment. These data suggest that HMB supplementation may promote hepatic total bile acid synthesis from cholesterol. To provide more evidence, we checked the expression of the *Cyp7a1* gene, which encodes a rate-limiting enzyme catalyzing cholesterol into bile acids [29], and found that *Cyp7a1* levels were significantly upregulated in the HMB supplementation group compared with the HFHCD control (Figure 4D), further indicating that HMB facilitates hepatic bile acid synthesis.

Next, we evaluated the beneficial effects of the elevated bile acid levels in the liver. Farnesoid X receptor (FXR) is a bile acid-activated nuclear receptor that plays a role in carbohydrate and lipid metabolism through transcriptional activation of target gene expression [30,31,32,33]. On account of this, we firstly checked *Fxr* expression and found that HMB supplementation significantly rescued HFHCD−suppressed *Fxr* expression (Figure 4E). Consistently, FXR activated−genes, *Ppara* and *Cpt1* [34], which participate in lipid oxidation, were also significantly rescued (Figure 4F,G). By contrast, lipogenic genes *Fasn* and *Acca* [34], which were inhibited by FXR and downregulated in the HFHCD group, were further blocked in the HMB−treated group (Figure 4H,I). Above all, these data suggest that HMB supplementation enhances hepatic lipid oxidation while it inhibits lipid synthesis via promotion of the conversion of cholesterol into bile acids, which is supposed to reduce lipid flux from the liver to adipose tissue and contribute to a reduction in fat deposition in layers.

### 3.4. Effects of Dietary HMB on Gut Microbial Diversity in HFHCD−Challenged Layer Chickens

**Figure 5:**
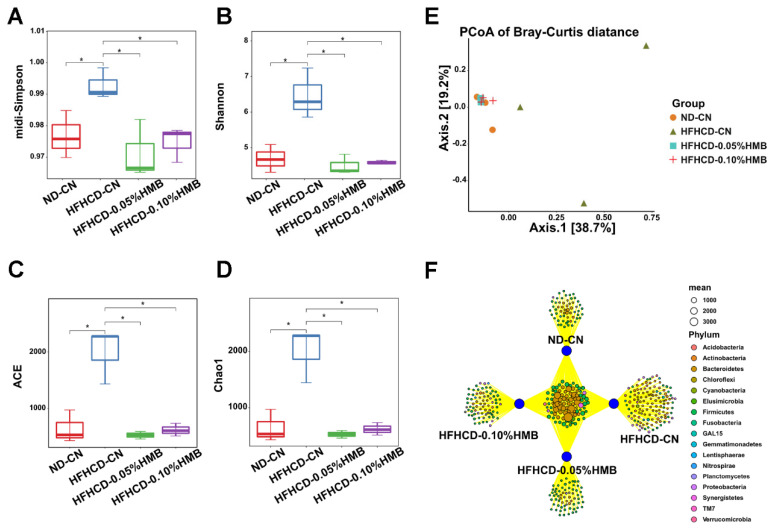
Effects of HMB on the richness and diversity of gut microflora in HFHCD–challenged layers. (**A**) Histogram for comparison of species diversity (midi-Simpson index). (**B**) Histogram for comparison of species diversity (Shannon index). (**C**) Histogram for comparison of species richness (ACE index). (**D**) Histogram for comparison of species richness (Chao1 index). (**E**) Principal coordinates analysis (PCoA). (**F**) Venn Diagram analysis. * *p* < 0.05.

The gut microbiota has been revealed to be highly involved in the process of fat deposition, and a growing number of studies have proven that exposure to certain exogenous compounds could affect its composition and function [35]. Therefore, we performed 16S rRNA sequencing to quantify the alterations in gut microbial populations among the four groups. First, we evaluated the alpha diversity of gut microbiota from the diversity (Simpson and Shannon) and richness (abundance-based coverage estimator (ACE) and Chao1). The results showed that HFHCD feeding significantly increased the diversity and richness of gut bacteria, while 0.05% and 0.10% HMB supplementation restored the diversity and richness to the basal level (Figure 5A–D). Then, a principal coordinates analysis (PCoA), which was based on Bray–Curtis similarity, was used to visualize the beta diversity of microbiota. The result showed that the samples from ND–CN and HFHCD–CN were in distinct clusters, but the dissimilarity between the two groups was significantly reduced and they were assigned to the same cluster after HMB supplementation (Figure 5E). Next, to explore the common and genotype–specific microbial communities among the four groups, Venn diagram analysis was applied, and the results showed that HFHCD feeding only altered a small proportion of bacterial flora, leaving large numbers of bacterial flora unaffected (Figure 5F). Interestingly, HMB supplementation led to the partial resumption of the alterations to microbial species caused by HFHCD feeding (Figure 5F). Above all, these data suggest that HMB supplementation restores HFHCD–disturbed gut microbial diversity.

### 3.5. Effects of Dietary HMB on Gut Microbial Composition in HFHCD–Challenged Layer Chickens

**Figure 6:**
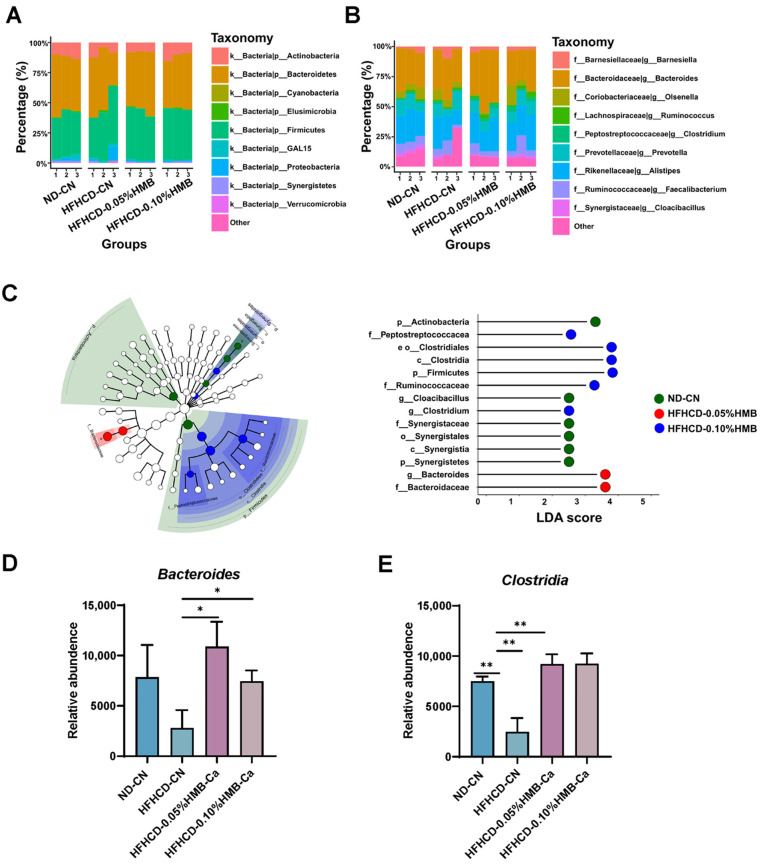
Effects of HMB on the composition of gut microbiota in HFHCD–challenged layers. (**A**) Microbiota composition at the phylum level. (**B**) Microbiota composition at the genus level. (**C**) The analysis of the non-parametric factors Kruskal–Wallis rank-sum test and LDA discrimination. (**D**) The abundance of Bacteroidetes in gut microbiota (*n* = 3). (**E**) The abundance of Clostridia in gut microbiota (*n* = 3). The results are shown as the means ± SDs; * *p* < 0.05, ** *p* < 0.01.

Next, we analyzed gut microbial composition in detail based on the abundance of bacteria. First, we found that, at the phylum level, the microbial communities in four groups were all dominated by *Actinobacteria*, *Bacteroidetes*, and *Firmicutes* (Figure 6A), while, at the genus level, the common predominant bacteria were *Bacteroides* and *Alistipes* (Figure 6B). Then, a linear discriminant analysis (LDA) effect size (LEfSe) analysis was performed to identify differentially abundant microbial populations among the four groups. The results showed that the dominant flora in the HFHCD−0.05% HMB group was *Bacteroidaceae* (*Bacteroides*), which was reported to be associated with leanness and other desirable health traits [36,37,38]. Interestingly, however, in the HFHCD−0.10% HMB group the predominant flora were *Clostridiales* (*Clostridia*) and *Firmicutes*, which have complex effects on fat deposition (Figure 6C) [39,40]. These differences implied that HMB level is an important effector in the modulation of gut microbiota. In line with the above findings, the HFHCD–suppressed abundance of *Bacteroides* and *Clostridia* were reversed by HMB supplementation (Figure 6D,E). Collectively, these data indicated that HMB supplementation countered the HFHCD–caused adverse alterations in the layers’ gut microbiota.

### 3.6. Effects of HMB on Gut Microbial Function in HFHCD–Challenged Layer Chickens

**Figure 7:**
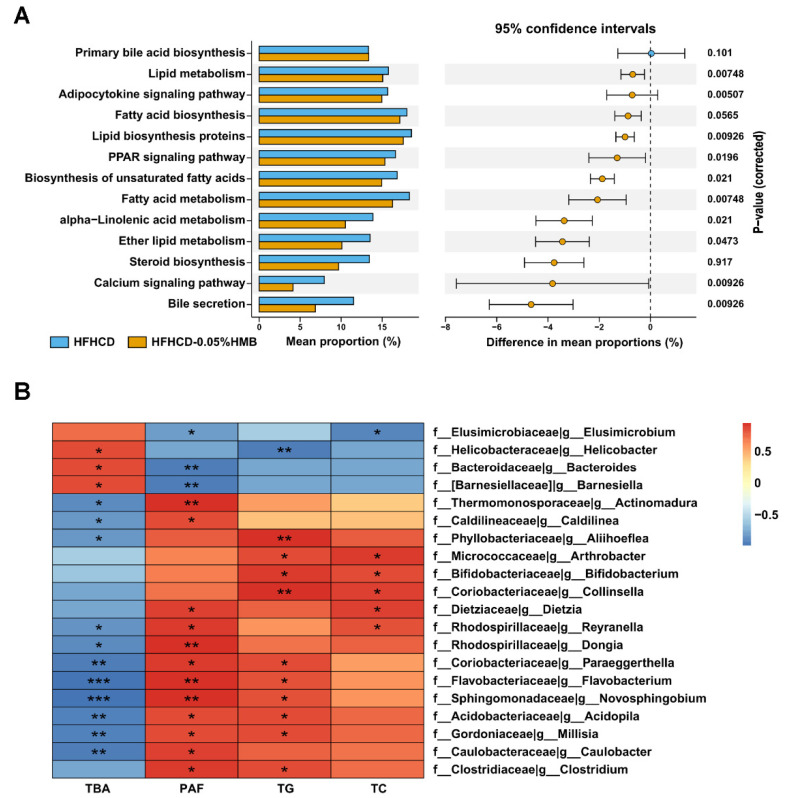
Effects of HMB on the function of gut microbiota in HFHCD–challenged layers. (**A**) KEGG functions of microbial communities in the HFHCD and HFHCD−0.05% group. Only lipid metabolism–related pathways are shown. (**B**) Heat map of correlation analyses of lipid metabolism–related parameters and relative abundances of gut microbiota. Only the top 20 significant correlations are shown. * *p* < 0.05, ** *p* < 0.01, *** *p* < 0,001. Abbreviations: TBA, total bile acids; PAF, percentage of abdominal fat; TG, triglycerides; TC, total cholesterol.

To uncover the potential functional interactions between gut microbiota and host, an analysis of KEGG functional orthologs was performed using the PICRUSt algorithm. As shown in Figure 7A, compared with the HFHCD–CN group, pathways related to lipid synthesis (e.g., steroid biosynthesis, biosynthesis of unsaturated fatty acids, and fatty acid biosynthesis) were markedly downregulated by 0.05% HMB supplementation. Consistent with the KEGG results, Pearson correlation analysis further revealed that there were significant associations between specific genera and metabolic phenotypes. For instance, commensal *Bacteroides* was positively correlated with the level of hepatic total bile acids (TBAs) but negatively correlated with the percentage of abdominal fat (PAF). Conversely, *Collinsella*, which has been linked to obesity, atherosclerosis, and inflammation [41], was positively correlated with PAF and TG but negatively correlated with TBA (Figure 7B). Collectively, these data indicate that gut microbiota partially mediated the beneficial effects of HMB supplementation on layer lipid metabolism.

## 4. Discussion

In laying hen husbandry, the excessive accumulation of fat and its associated metabolic disorders are considered one of the major causes of reduced egg production [1]. Thus, it is urgent to seek drugs or feed additives to improve fat metabolism in laying hens. While HMB, a metabolite of L-leucine in the liver, was recently reported to modulate hepatic lipid metabolism in broiler chickens [22], it is still unknown whether dietary supplementation with HMB could alleviate fat over-accumulation in laying hens. This is because there are huge differences between broiler chickens and layer chickens in terms of genetics, nutrition, physiology, and feeding management [42]. In particular, broilers are selected for meat production and can be slaughtered as early as around 35 days of age to maximize profits [43]. By contrast, layers are used for egg production and can be kept for 2–3 years [44]. As such, laying hens are more prone to developing fat-related metabolic dysfunctions than broiler chickens. Therefore, it is of great significance to investigate whether HMB has fat-lowering effects in layers. In this study, we found that dietary supplementation with HMB significantly ameliorated HFHCD–induced fat deposition in hens, indicating that HMB is a good candidate as a new feed additive in the laying hen industry.

Compared with broiler chickens, one obvious difficulty in studying laying hens is that layers have a long laying cycle; thus, studies take more time and incur higher costs than studies on broilers. To overcome this difficulty, we established a high-fat and high-cholesterol (13% lard + 2% cholesterol) diet (HFHCD)-induced fat over-accumulated growing layer model to mimic naturally occurring obesity in old laying hens. We found that 6 weeks of HFHCD feeding of 1-month-old growing hens was sufficient to induce remarkable abdominal fat deposition (Figure 2); however, it was not sufficient to cause fatty liver (Figure 3), suggesting that the obese model was successful. Since it is believed that fatty liver is one consequence of obesity [45], in the future, prolonged HFHCD feeding may be used as a strategy to establish a laying hen model of fatty liver.

By using the HFHCD–induced fat over-accumulation model, we found that HMB supplementation plays a fat-lowering role in layers (Figure 2 and Figure 3). This finding is consistent with previous reports on mice and pigs [19,20], suggesting that the function of HMB in lipid metabolism is conserved from birds to mammals. Furthermore, we found that gut microbiota partially mediate the protective effects of HMB against fat accumulation (Figure 5, Figure 6 and Figure 7). Frankly, this is an expected finding because the gut microbiota is well-known for its broad range of roles in the regulation of many physiological and pathological processes, particularly metabolism [46]. In addition, studies in mice and broilers have already indicated that the gut microbiota plays a role in the lipid-lowering action of HMB [20,22]. However, until now, it has been unclear how HMB shapes the gut microbiota. Thus, future studies are needed to elucidate the underlying mechanisms that may deepen our knowledge and provide insights into developing new drugs or feed additives.

Meanwhile, unexpectedly, we revealed another mechanism—the bile acid—regulated lipid metabolism pathway in the liver. While it was already known that hepatic bile acid plays an important role in the regulation of lipid metabolism [47], it is still unknown whether bile acid is involved in the beneficial effects of HMB. Here, we found that HMB facilitates the conversion of cholesterol to bile acids in the liver, which results in reduction in cholesterol levels but increase in bile acid levels in the liver (Figure 3A,C). In line with the elevated levels of hepatic bile acids and the nuclear receptor *Fxra*, the lipid oxidation genes *Ppara* and *Cpt1* were upregulated, whereas the lipogenenic genes *Fasn* and *Acca* were downregulated (Figure 4E–I) [34]. On account of this, the output of lipid flux from liver to adipose tissue could be remarkably reduced, ameliorating fat deposition in layers. Mechanistically, we found that HMB upregulated the expression of cholesterol 7alpha-hydroxylase (CYP7A1), a key enzyme in bile acid synthesis from cholesterol (Figure 3D). Currently, it is unknown how HMB regulates hepatic *Cyp7a1* expression, which could be studied in future work.

Although our work has demonstrated that dietary supplementation with HMB facilitates lipid catabolism and protects layers against fat over-accumulation, it is still unknown whether HMB improves metabolic health, egg quality, as well as egg production in laying hens under standard poultry farming conditions. Furthermore, it should be noted that the beneficial roles of HMB are highly dose- and species-dependent and that lower doses seem to have better effects than higher doses [19,20,22]. However, the optimal doses of HMB for different species of birds and animals are as yet undetermined. Therefore, more studies are required to tackle these unanswered questions in the future.

In summary, this study investigated the effects and underlying mechanisms of dietary HMB supplementation on fat deposition in layers using a HFHCD–induced obese bird model. The results have shown that 0.05% or 0.10% HMB addition significantly ameliorates HFHCD–induced abdominal fat deposition, probably via enhancing hepatic bile acid synthesis and shaping the gut microbiota. The findings in this study provide a new therapeutic strategy for preventing and tackling fat–associated metabolic disorders in the poultry industry.

## Figures and Tables

**Table 1:** Composition and nutrient levels of basal diets (air-dry basis) (%).

Items	Content	
	1 to 4 Weeks	4 to 10 Weeks
Ingredients, %		
Corn	56.80	51.50
Wheat bran	5.00	10.00
Soybean meal	30.00	32.00
Fish meal	3.00	-
Limestone	1.40	1.50
CaHPO_4_	1.20	1.00
Lard oil	0.60	2.00
Premix ^1^	2.00	2.00
Total	100.00	100.00
Nutrients (calculated value)		
CP	20.01	20.70
EE	3.44	4.42
Ash	5.26	5.12
Ca	1.27	1.28
TP	0.60	0.55
AP	0.73	0.78
Lys	1.10	1.03
Met	0.48	0.43

^1^ The premix provided the following per Kg of diets: VB1 3.5 mg, VB2 8.25 mg, VB6 6.75 mg, VB12 0.040 mg, VA11500 IU, VD3 3200 IU, VK3 2.5 mg, Cu 10.8 mg, Fe 95 mg, Mn 90 mg, Zn 80 mg, Se 0.28 mg, pantothenic acid 21 mg, nicotinic acid 35 mg, folic acid 1.25 mg, I 0.38 mg, Co 0.1 mg, Choline 650 mg, biotin 0.30 mg.

**Table 2:** Primer sequences for each target gene.

Genes	Primer Sequences (5′→3′)	GeneBank Accession Number
*Accα*	F: AGTCCTGATTGAGCATGGCA	NM_205505.1
	R: CTCCAGATGGCGGTAGATTC	
*β-actin*	F: TGCGTGACATCAAGGAGAAG	NM_205518.1
	R: TGCCAGGGTACATTGTGGTA	
*Cpt1*	F: GCCAAGTCGCTCGCTGATGAC	DQ314726.1
	R: ACGCCTCGTAGGTCAGACAGAAC	
*Cyp7a1*	F: CATTCTGTTGCCAGGTGATGTT	AY700578
	R: GCTCTCTCTGTTTCCCGCTTT	
*Fasn*	F: TGAAGGACCTTATCGATTGC	NM_205155.4
	R: GCATGGGAAGCATTTTGTTGT	
*Fxr*	F: AGTAGAAGCCATGTTCCTCCGTT	AF492497
	R: GCAGTGCATATTCCTCCTGTGTC	
*Ppara*	F: GAATGCCACAAGCGGAGAAGGAG	NM_001001464.1
	R: GCTCGCAGATCAGCAGATTCAGG	

## Data Availability

All data are presented in the main manuscript.

## Abbreviations

Accα	Acetyl-coa carboxylase alpha
ADFI	Average daily feed intake
ADG	Average daily gain
ASV	Amplicon sequence variant
Cpt1	Carnitine palmitoyltransferase 1
Cyp7a1	Cytochrome P450 family 7 subfamily A member 1
DNL	De novo lipogenesis
F/G	Feed-to-gain ratio
Fasn	Fatty acid synthase
FLHS	Fatty liver haemorrhagic syndrome
Fxr	Farnesoid X-activated receptor
HFD	High-fat diet
HFHCD	High-fat and high-cholesterol diet
HMB	Β-hydroxy-β-methylbutyrate
LDA	Linear discriminant analysis
LEfSe	Linear discriminant analysis (LDA) effect size
NAFLD	Non-alchoholic fatty liver disease
PAF	Percentage of abdominal fat
Ppara	Peroxisome proliferator-activated receptor alpha
SCFAs	Short-chain fatty acids
TBA	Total bile acid
TC	Total cholesterol
TG	Triglycerides
VLDL	Very low-density lipoprotein
Vg	Vitellogenin
